# On the Chlorosis of Adults

**Published:** 1846-08

**Authors:** 


*On the Chlorosis of Adults.* By M. Blaud.—"There is a disease pretty commonly met with, which seems to me to have escaped the observation of most practitioners. This affection, of great danger if misunderstood, is the *Chlorosis of Adults.* I do not allude to that decoloration of the skin dependent upon some prior disease, such as hemorrhage, intermit-

* It appears from the Boston Medical Journal, for January, 1846, that a Dr. Waterman of Buffalo, N. Y. has been sentenced to hard labor for three years, in the State Prison, for *disinterring* a dead body for dissection! Has the age of Vesalius returned? Is it a crime to increase human happiness by increasing human knowledge, its indispensable element? Shall the worms on land, and the sharks at sea, be entrusted with the whole knowledge of Anatomy? Is that true philanthropy which seeks to save the ravisher and murderer from capital punishment, that he may be placed in prison side by side with an honest men, whose only crime consists in operating on the dead, that he may know how to operate on the living with safety? The Quack is privileged to kill with impunity! Will he be in haste to change his ways—to become a good citizen—that is, to get a knowledge of his profession, when the punishmens of a felon is to be the reward of his pains?

tent fever, or some organic lesion, acute or chronic, &c.; but an *essential idiopathic chlorosis*, proceeding from no other cause but such as produce it directly, that is, a particular defect of hæmatosis, and which can only be relieved by re-establishing the normal condition of this function.

"Chlorosis is by no means peculiar to young girls arrived at puberty, and it is from want of attention to this fact, tht many practitioners have mistaken it, when occuring in adults, for different organic lesions, which in fact had no existence. Some, seeing the pale or yellow skin, have declared it to be symptomatic of hepatic lesion, and others have attributed it to affection of the spleen. Some attribute the dyspnœa and palpitations to lesions of the heart or large vessels, and others see in these the symptoms of an asthma. Such different views lead to various plans of treatment, none of which are efficacious, the chlorosis, indeed seeming to derive a new intensity from their operation. Chlorosis may attack adults of either sex, any age, and of any condition in life. We have seen subjects of it in persons leading the most active and laborious lives, and others passing their time in luxurious idleness. The sober and the intemperate; those whom misery oppresses, and those who enjoy every abundance in life, may alike become its victims. So that the *predisposing* causes of the affection are as yet very obscure. The colourless and serous condition of the blood, the general debility and languor of every function, seem to show that defective hæmatosis is the *proximate* cause. The *symptoms* are the same as in the chlorosis of young girls—such as the yellowish-green skin (the conjunctivæ preserving their normal whiteness.) dyspnœa upon locomotion, *bruit de souffle* in the course of the carotids, &c., Still there are modifications noticeable. The colour of the skin is rather greyish or earthy, than yellow, in consequence of its rough or wrinkled state, especially in the face. The palpitations are more severe; while there is moreover a deep, insupportable *malaise*, sometimes accompanied by a disposition to suicide, and a more or less abundant anal hemorrhage coming on at irregular intervals. With the general langour and disorder of the digestive organs, there is œdema of the extremities; and, if the disease be left to itself, a serous effusion into the cavity of the abdomen results from the atony of the absorbents. However severe these symptoms may appear, they never resist appropriate *treatment*, when this is not resorted to too late."

Of the cases he has met with, M. Blaud selects eight for description, four of them being women from 26 to 37 years of age, and the others, men engaged in active or laborious occupations. In most of these, various kinds of treatment had been employed before they came under the author's care, in the idea that organic disease was present: but he, guided by the general appearance of the patient and the absence of marked signs of such lesions, treated them all for chlorosis with complete success. He employed his own anti-chlorotic pills,* which have attained so high a reputation in France, in every case; and complete cures resulted in periods varying from 10 to 30 days of taking them. He adds in a note,

* M. Blaud's pills are thus composed and administered. Let equal parts of sulphate of iron and sub-carbonate of potass be finely powdered separately, and then intimately mixed. Beat them into a mass with tragacanth mucilage. Twelve grains form two pills. Take two morning, mid-day and evening, on the first, second, and third days: four morning and evening on the fourth, fifth and sixth days; four morning, mid-day, and evening, on the seventh, eigth, and ninth days; six morning and evening on the tenth, eleventh and twelfth days: and then six morning, noon and evening, until cured.

that long experience has convinced him that these pills are also a specific in *intermittent fever accompanied with œdema of the lower extremities*, which has resisted quinine and other febrifuges.—*Medico-Chirur. Review.*

